# Evaluation of Ceftaroline Use in Pediatric Patients: A Retrospective Case Series

**DOI:** 10.3390/antibiotics14090864

**Published:** 2025-08-28

**Authors:** Amy Miller, Madison Grizzle, Halee Van Poppel, Gustavo R. Alvira-Arill, Richard Lueking, Stephen A. Thacker, Krutika Mediwala Hornback, Taylor Morrisette

**Affiliations:** 1 Department of Pharmacy Services, Medical University of South Carolina Health, Charleston, SC 29425, USA; amy.miller1@vcuhealth.org (A.M.); alviraar@musc.edu (G.R.A.-A.); mediwala@musc.edu (K.M.H.); 2 Department of Clinical Pharmacy & Outcomes Sciences, Medical University of South Carolina College of Pharmacy, Charleston, SC 29425, USA; grizzle@musc.edu (M.G.); vanpoppe@musc.edu (H.V.P.); 3 Department of Internal Medicine, Division of Infectious Diseases, Medical University of South Carolina Health, Charleston, SC 29425, USA; lueking@musc.edu; 4 Department of Pediatrics, Division of Infectious Diseases, Medical University of South Carolina Health, Charleston, SC 29425, USA; thackest@musc.edu

**Keywords:** adolescent, child, ceftaroline, methicillin-resistant *Staphylococcus aureus*, pediatrics

## Abstract

**Background/Objectives**: Ceftaroline (CPT) is a broad-spectrum, fifth-generation cephalosporin with in vitro activity against methicillin-resistant *Staphylococcus aureus* (MRSA) and drug-resistant *Streptococcus pneumoniae*. Real-world data on its use in pediatric patients remain limited. This study aimed to the describe clinical characteristics and outcomes associated with CPT use in pediatric patients at a pediatric academic medical center. **Methods**: This retrospective case series evaluated patients under 18 years of age who received CPT between November 2016 and August 2023. The primary outcome was clinical success, defined as a composite of 30-day survival, absence of microbiological recurrence within 30 days, and/or resolution of acute infection signs and symptoms without therapy modification due to clinical failure. The secondary outcomes included adverse effects potentially attributable to CPT and the clinical rationale guiding its use. **Results**: Among 25 patients, most were male (68%) with a median (IQR) age of 3.4 (1.4–14.3) years. The indications for use commonly included respiratory infections (48%), bacteremia (16%), and/or skin and soft tissue (12%) infections. The frequently used dosing regimens included 12 mg/kg (36%) and 8 mg/kg (28%) q8hr, with a median (IQR) duration of therapy of 4.6 (1.7–10.0) days. Clinical success was achieved in 96% of patients. No adverse effects attributable to CPT were observed and CPT was commonly used for escalation (40%) and/or issues with alternative therapies (36%). **Conclusions**: CPT use was associated with high clinical success rates and no observed adverse effects in this pediatric report. These findings support its use as a therapeutic option when the alternatives are limited. Larger multicenter studies are needed to further evaluate the clinical outcomes and safety of CPT use in pediatric patients.

## 1. Introduction

Antimicrobial resistance (AMR) represents a mounting global health crisis, and its impact on pediatric populations is increasingly evident. Younger children remain particularly vulnerable, with infectious diseases (IDs) and their complications continuing to be a leading cause of morbidity and mortality worldwide [1,2]. Despite advances in antimicrobial development, pathogens such as methicillin-resistant *Staphylococcus aureus* (MRSA) and drug-resistant *Streptococcus pneumoniae* (DRSP) continue to pose significant risks to both adult and pediatric patients and are both classified as serious threats by the Centers for Disease Control and Prevention (CDC) [3,4,5,6]. Although vancomycin remains a first-line parenteral agent for invasive infections, its use is complicated by the need for therapeutic drug monitoring to ensure efficacy and to minimize nephrotoxicity [7]. Second-line agents, such as daptomycin and linezolid, are also associated with notable adverse drug reactions and other limitations [6,8,9,10,11]. These challenges underscore the urgent need for pediatric-specific data to guide antimicrobial selection, dosing strategies, and clinical outcome optimization in the treatment of resistant Gram-positive infections [6].

Ceftaroline fosamil, the prodrug of ceftaroline (CPT), represents a paradigm shift within the beta-lactam class as a fifth-generation cephalosporin exhibiting a uniquely broad-spectrum of activity, including against MRSA [12,13,14,15,16]. CPT’s distinctiveness is attributed to its unique structure, which enables binding to penicillin-binding protein 2a (PBP2a), the modified PBP responsible for conferring beta-lactam resistance in MRSA [12]. CPT also demonstrates potent in vitro activity against DRSP, some Enterobacterales, *Haemophilus influenzae*, and *Moraxella catarrhalis* [12,14,15,16]. Approved by the United States (U.S.) Food and Drug Administration (FDA) in 2010, CPT was initially indicated in adults for acute bacterial skin and skin structure infections (ABSSSIs) and community-acquired bacterial pneumonia (CABP). Its pediatric indications followed in 2016 and 2019, extending its use to children at least 34 weeks gestational age and 12 days postnatal age for ABSSSIs and two months of age and older for CABP [17]. Since its approval, CPT use in pediatric patients has progressively increased due to its potent in vitro activity and favorable efficacy and safety compared to conventional first- and second-line agents [12,13,14,15,16,17,18,19,20,21,22].

While extensive real-world evidence supports CPT use in adults, the data characterizing its utilization and clinical success in pediatric patients remain limited [19,20,21,22,23,24,25,26]. Therefore, this study aimed to characterize contemporary CPT use, including the clinical features and treatment outcomes of pediatric patients treated with CPT.

## 2. Results

**Figure 1:**
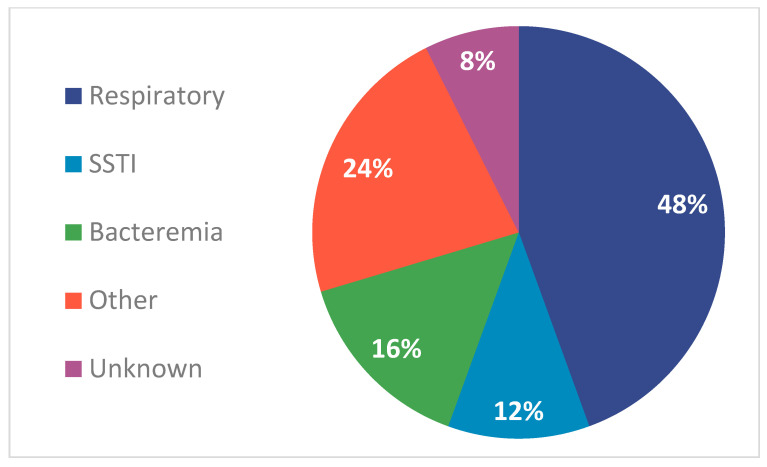
Infection diagnoses. SSTI: skin and soft-tissue infection; Data > 100%, as some patients had mixed infections; 75% of patients with bacteremia had infections that were classified as central line-associated bloodstream infections; “Other” infections included those such as disseminated *Mycobacterium abscesses* infection, presumed endocarditis, and ventriculitis.

A total of 25 patients were included in this evaluation, who were predominantly white (11/25, 44%) and male (17/25, 68%), with the median (IQR) age of patients being 3.4 (1.4–14.3) years. Most of the patients were admitted to the intensive care unit (ICU), with 14/25 (56%) receiving care in the pediatric intensive care unit. Notably, 15/25 (60%) patients had at least one risk factor for MRSA, with prior antibiotic exposure within 12 months and long-term central venous access within 12 months being the most common, both present in 11/25 (44%) patients. Respiratory infections constituted the most frequent primary diagnosis (12/25, 48%), followed by bacteremia (4/25, 16%) and skin and soft-tissue infections (3/25, 12%). Source control procedures were performed and achieved in nearly half of the population (11/25, 44%), including debridement in 3/25 (12%) and incision and drainage procedures in 2/25 (8%). All the baseline characteristics and diagnoses can be found in Table 1 and Figure 1, respectively.

**Figure 2:**
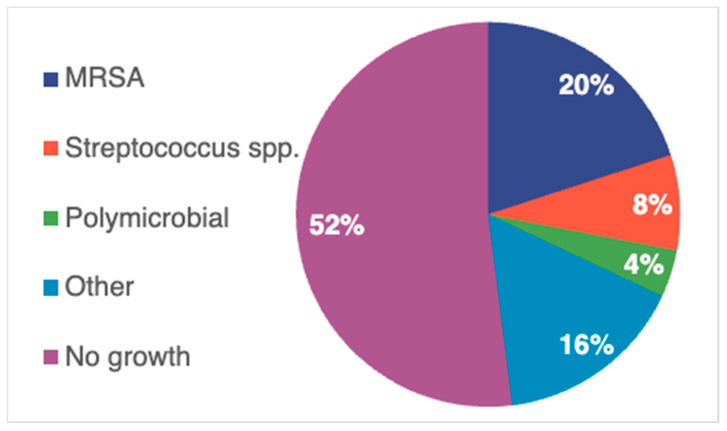
Microbiological data. MRSA: methicillin-resistant *Staphylococcus aureus*; n = 25.

Among those patients with positive cultures, MRSA and *Streptococcus* species were commonly isolated organisms (Figure 2). Antimicrobial susceptibility testing revealed a vancomycin minimum inhibitory concentration (MIC) of 1 mg/L in all MRSA isolates, while CPT susceptibility was only reported for a single MRSA isolate that exhibited an MIC of 0.5 mg/L.

All the patients received a consultation from pediatric ID, with most CPT initiations (22/25, 88%) being recommended by pediatric ID. Prior to CPT administration, 22/25 (88%) patients had been treated with other antibiotics and the median (IQR) duration of antibiotic therapy prior to CPT initiation was 73.0 (20.9–139.8) hours. Most patients received CPT 12 mg/kg q8h (9/25, 36%) or 8 mg/kg q8h (7/25, 28%) for a median (IQR) total treatment duration of 4.6 (1.7–10.0) days. All other infection and treatment characteristics can be found in Table 2.

**Figure 3:**
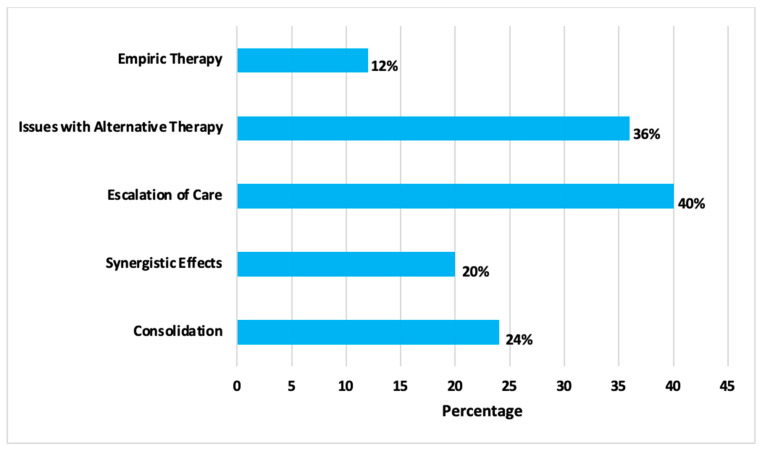
Rationale for ceftaroline use. Data > 100%, as some patients received CPT for >1 reason.

The primary outcome of composite clinical success was achieved in most patients (24/25, 96%), with a single case of 30-day mortality in a patient that underwent a previous hematopoietic stem cell transplantation with bacteremia caused by *Rothia mucilaginosa* who received combination antibiotic therapy. Furthermore, no adverse events attributable to CPT were identified during the study period. The clinical rationale for CPT utilization is shown in Figure 3.

## 3. Discussion

This retrospective, real-world study evaluated the clinical use and associated outcomes in children treated with CPT for serious infections. These findings demonstrate that CPT was associated with high rates of clinical success and was found to be safe. These results reinforce CPT’s emerging role as an antimicrobial option in children, particularly when historical first-line agents are limited by toxicities, drug–drug interactions, contraindications, and/or clinical failure.

Vancomycin remains the gold-standard first-line therapy for most invasive Gram-positive infections in both adult and pediatric patients [27,28,29]. However, complications of vancomycin include nephrotoxicity, infusion-related reactions, and the need for therapeutic drug monitoring [28]. Daptomycin represents an effective alternative to vancomycin; however, its use in certain populations is constrained by several factors, including its inactivation by pulmonary surfactant, rendering it ineffective for the treatment of pneumonia, as well as the risk of myopathy, which necessitates the regular monitoring of creatine phosphokinase levels [8]. Although linezolid is frequently employed in the management of resistant Gram-positive infections, its use is constrained by adverse effects linked to prolonged therapy, as well as some clinician hesitation regarding its bacteriostatic activity and potential interactions with serotonergic medications [9,10,11]. These limitations underscore the need for efficacious alternatives with more favorable profiles.

Ceftaroline has demonstrated potent in vitro activity with high rates of susceptibility noted against various Gram-positive organisms, including MRSA and DRSP [15,16]. Importantly, previous population pharmacokinetic modeling studies have further supported CPT’s use in pediatrics, with comparable exposure profiles to adult regimens [30,31]. Furthermore, several pediatric trials have evaluated CPT use, including in ABSSSI and CABP. These trials, which included children aged 2 months to 17 years, demonstrated similar efficacy and safety outcomes between CPT and comparator agents [32,33,34].

Although CPT has been studied in children with cystic fibrosis, robust real-world data in the broader pediatric population remain notably limited [19,20,21,22]. In one retrospective chart review involving 180 cystic fibrosis patients (50% receiving CPT) aged 0 to 21 years, CPT was compared to vancomycin, with no significant differences observed between the two agents in terms of mean change in forced expiratory volume in one second (FEV_1_) or hospital readmissions [19]. In another study of 16 pre-term infants with late-onset staphylococcal sepsis that failed vancomycin therapy, clinical success rates were approximately 70%, with no severe adverse drug reactions reported [20]. In contrast, there are numerous, large, real-world studies in adult populations that have provided supportive evidence of CPT’s effectiveness in treating complicated ABSSSIs, pneumonia, and bloodstream infections, with high clinical response rates observed [23,24,25,26].

Although limited by its retrospective design, single-center setting, and absence of a comparator group, this study offers valuable insights into the high clinical success rates, safety, and current prescribing practices of CPT at a pediatric academic medical center. Other limitations include potential overestimation of clinical success due to untracked readmissions at external facilities, the lack of a minimum CPT duration of therapy, and relatively high rates of combination antimicrobial therapy, which may have inflated the attribution of positive outcomes to the therapy. Furthermore, only one isolate had formal CPT susceptibility testing, yet high clinical success rates were still achieved, which is consistent with the data showing CPT’s potent in vitro activity against commonly encountered drug-resistant Gram-positive organisms [15,16]. Despite these findings, the lack of real-world pediatric studies assessing CPT use outside of clinical trials remains a significant gap. This study contributes novel data on CPT utilization patterns, clinical decision-making, and outcomes in children with serious infections.

## 4. Materials and Methods

This was a retrospective case series of pediatric patients (age < 18 years) that was conducted at the Medical University of South Carolina (MUSC) Health. Pediatric patients who received CPT at the Children’s Hospital between November 2016 and August 2023 were eligible for inclusion. The decision to initiate CPT, as well as its duration, was made at the discretion of the treating clinician(s), based on the presumed overall clinical picture, as well as the suspected or confirmed pathogen(s) and infectious source(s). The dosing of CPT was based off the prescribing information, adjusted for age, weight, renal function, and indication, at the discretion of the treating provider(s) [17].

The primary outcome was a composite measure of clinical success, defined by 30-day survival, the absence of microbiologic recurrence within 30 days, and/or the resolution of acute infection signs and symptoms without modifications in therapy due to clinical failure. Modifications for de-escalation or oral step-down therapy were not considered clinical failures. The secondary outcomes included adverse effects and the clinical rationale regarding CPT use. Adverse effects were identified and recorded through review of clinical documentation in the patients’ medical records. Additional variables potentially related to the outcomes were collected and included gender, age, weight, race, intensive care unit admission, MRSA risk factors, vital signs and laboratory values at index culture or initial antibiotic administration (whichever occurred sooner), and hospital length of stay [35,36,37]. The treatment-related variables included prior antibiotic use, CPT dosing and duration, concomitant antibiotic use, and CPT discharge prescriptions.

The rationale for CPT use was categorized based on the documentation provided by the treating clinician(s) and/or the clinical judgement of data abstractors at the time of review. Empiric therapy was defined as the administration of CPT for the treatment of a suspected infection; issues with alternative therapy included variables such as toxicity, drug interactions, and/or contraindications; escalation of care was defined as initiation of CPT in the setting of presumed failure of empiric or targeted therapy; use for potential synergistic effects was based off in vitro data; and consolidation of therapy included when CPT was selected as a continuation agent following initial therapy for streamlining purposes and therapeutic simplification [38,39].

Proportions were used to describe the categorical variables and medians with IQRs were used for the continuous variables. Statistical analyses were performed using Microsoft Excel (version 2502; Microsoft Corporation; Redmond, Washington, DC, USA). The study protocol was submitted to the local institutional review board and was determined to be exempt from full board review.

## 5. Conclusions

CPT appears to be a well-tolerated and promising therapeutic option in pediatric patients, particularly when conventional therapies are not viable or ideal. Further multicenter, comparative studies are essential to better define the role of CPT in pediatric practice, guide stewardship efforts, and inform evidence-based prescribing in this vulnerable population.

## Figures and Tables

**Table 1:** Baseline characteristics.

Parameters	n = 25
Male	17 (68)
Age, years, median (IQR)	3.4 (1.4–14.3)
Age < 2 years	8 (32)
Age 2–11 years	10 (40)
Age 12–17 years	7 (28)
Weight, kg, median (IQR)	15.7 (10.7–54.4)
Race	
White	11 (44)
Black	9 (36)
ICU Admission	
PICU	14 (56)
PCICU	1 (4)
NICU	1 (4)
MRSA Risk Factors	
Hospitalization within 12 months	9 (36)
Antibiotic exposure within 12 months	11 (44)
MRSA colonization or infection within 12 months	0 (0)
Invasive procedures within 12 months	6 (24)
Long-term central venous access within 12 months	11 (44)
Prior ABSSSI within 12 months	0 (0)
Vital Signs and Laboratory Values *, median (IQR)	
Temperature, °Celsius	37.2 (36.7–37.9)
HR, beats per minute	126.0 (115.0–155.0)
RR, breaths per minute	26.0 (24.0–36.0)
WBC, ×10^9^/L	8.2 (6.2–17.0)
CRP, mg/L ^	12.8 (11.8–17.2)
Procalcitonin, ng/mL ^^	0.4 (0.3–3.9)
SCr, mg/dL ^^^	0.5 (0.3–0.7)

Data reported as n (%) unless otherwise noted. * Time of index culture collection or antibiotic initiation (whichever occurred sooner). ^ Data reported for seven patients. ^^ Data reported for six patients. ^^^ Data reported for 23 patients. IQR: interquartile range; kg: kilograms; ICU: intensive care unit; PICU: pediatric ICU; PCICU: pediatric cardiac ICU; NICU: neonatal ICU; MRSA: methicillin-resistant *Staphylococcus aureus*; ABSSSI: acute bacterial skin and skin structure infection; HR: heart rate; RR: respiratory rate; WBC: white blood cell count; CRP: C-reactive protein; SCr: serum creatinine.

**Table 2:** Infection and treatment characteristics.

Parameters	n = 25
Hospital length of stay, days, median (IQR)	10.0 (7.8–34.8)
Pediatric ID consult	25 (100)
CPT recommended from pediatric ID	22 (88)
Antibiotics received prior to CPT initiation	22 (88)
Total duration of antibiotics prior to CPT initiation (hours), median (IQR)	73.0 (20.9–139.8)
CPT 12 mg/kg q8h	9 (36)
CPT 8 mg/kg q8h	7 (28)
Duration of CPT, days, median (IQR)	4.6 (1.7–10.0)
Concomitant antibiotics	16 (64)
CPT at discharge	4 (16)

ID: infectious disease; IQR: interquartile range; CPT: ceftaroline.

## Data Availability

Data can be made available upon request.

## References

[1] Romandini A., Pani A., Schenardi P.A., Pattarino G.A.C., De Giacomo C., Scaglione F. (2021). Antibiotic resistance in pediatric infections: Global emerging threats, predicting the near future. Antibiotics.

[2] World Health Organization (2022). Shaping the Global Innovation and Access Landscape for Better Paediatric Medicines.

[3] Centers for Disease Control and Prevention (2019). Antibiotic Resistance Threats in the United States.

[4] Prochaska E.C., Xiao S., Tamma P.D., Sick-Samuels A., Schumacher C., Gadala A., Carroll K.C., Milstone A.M. (2023). Trends in pediatric community-onset *Staphylococcus aureus* antibiotic susceptibilities over a five-year period in a multihospital health system. Antimicrob. Steward. Healthc. Epidemiol..

[5] Gerber J.S., Coffin S.E., Smathers S.A., Zaoutis T.E. (2009). Trends in the incidence of methicillin-resistant Staphylococcus aureus infection in children’s hospitals in the United States. Clin. Infect. Dis..

[6] Chiusaroli L., Liberati C., Rulli L., Barbieri E., De Pieri M., Di Chiara C., Mengato D., Giaquinto C., Donà D. (2023). Therapeutic options and outcomes for the treatment of children with gram-positive bacteria with resistances of concern: A systematic review. Antibiotics.

[7] Rybak M.J., Le J., Lodise T.P., Levine D.P., Bradley J.S., Liu C., Mueller B.A., Pai M.P., Wong-Beringer A., Rotschafer J.C. (2020). Therapeutic monitoring of vancomycin for serious methicillin-resistant *Staphylococcus aureus* infections: A revised consensus guideline and review by the American Society of Health-System Pharmacists, the Infectious Disease Society of America, The Pediatric Infectious Diseases Society, and the Society of Infectious Diseases Pharmacists. Am. J. Health System Pharm..

[8] (2023). Daptomycin [Package Insert].

[9] (2013). Linezolid [Package Insert].

[10] Clemett D., Markham A. (2000). Linezolid. Drugs.

[11] Woytowish M.R., Maynor L.M. (2013). Clinical relevance of linezolid-associated serotonin toxicity. Ann. Pharmacother..

[12] Duplessis C., Crum-Cianflone N.F. (2011). Ceftaroline: A new cephalosporin with activity against methicillin-resistant *Staphylococcus aureus* (MRSA). Clin. Med. Rev. Ther..

[13] Croisier-Bertin D., Piroth L., Charles P.E., Larribeau A., Biek D., Ge Y., Chavanet P. (2011). Ceftaroline versus ceftriaxone in a highly penicillin-resistant pneumococcal pneumonia rabbit model using simulated human dosing. Antimicrob. Agents Chemother..

[14] Biedenbach D.J., Iaconis J.P., Sahm D.F. (2016). Comparative in vitro activities of ceftaroline and ceftriaxone against bacterial pathogens associated with respiratory tract infections: Results from the AWARE surveillance study. J. Antimicrob. Chemother..

[15] Kempf M., Arhin F.F., Kuraieva A., Utt E. (2024). In vitro activity of ceftaroline against isolates of gram-positive bacteria from patients with bloodstream infections collected as a part of ATLAS between 2017 and 2020. Infect. Drug Resist..

[16] Utt E., Kantecki M., Cabezas-Camarero G., Esposito S. (2023). Evaluation of in vitro activity of ceftaroline against pathogens associated with community-acquired pneumonia: ATLAS program 2017–2019. J. Glob. Antimicrob. Resist..

[17] (2024). Ceftaroline [Package Insert].

[18] Valentino M.S., Borgia P., Deut V., Lorenzi I., Barabino P., Ugolotti E., Mariani M., Bagnasco F., Castagnola E. (2023). Changes in the Use of Antibiotics for Methicillin-Resistant *Staphylococcus* aureus Bloodstream Infections in Children: A 5-Year Retrospective, Single Center Study. Antibiotics.

[19] Branstetter J., Searcy H., Benner K., Yarbrough A., Crowder C., Troxler B. (2020). Ceftaroline vs vancomycin for the treatment of acute pulmonary exacerbations in pediatric patients with cystic fibrosis. Pediatr. Pulmonol..

[20] Callies A., Martin-Perceval L., Crémet L., Gély L., Ruellan A.-L., Verdier M.-C., Gregoire M., Flamant C., Guillouzouic A., Prot-Labarthe S. (2023). Safety and efficacy of ceftaroline in neonates with staphylococcal late-onset sepsis: A case series analysis. Pediatr. Infect. Dis. J..

[21] Cies J.J., Moore W.S., Enache A., Chopra A. (2018). Ceftaroline for suspected or confirmed invasive methicillin-resistant *Staphylococcus aureus*: A pharmacokinetic case series. Pediatr. Crit. Care Med..

[22] Williams A.W., Newman P.M., Ocheltree S., Beaty R., Hassoun A. (2015). Ceftaroline fosamil use in 2 pediatric patients with invasive methicillin-resistant *Staphylococcus aureus* infections. J. Pediatric Pharmacol. Ther..

[23] Soriano A., Bassetti M., Gogos C., Ferry T., de Pablo R., Ansari W., Kantecki M., Schweikert B., Luna G., Blasi F. (2024). Ceftaroline fosamil treatment patterns and outcomes in adults with community-acquired pneumonia: A real-world multinational, retrospective study. JAC Antimicrob. Resist..

[24] Ferry T., Gogos C., Soriano A., Blasi F., Ansari W., Kantecki M., Schweikert B., Luna G., Bassetti M. (2024). Real-world use and treatment outcomes of ceftaroline fosamil in patients with complicated skin and soft tissue infection: A multinational retrospective study. Infect. Drug Resist..

[25] de la Villa S., Escrihuela-Vidal F., Fernández-Hidalgo N., Escudero-Sánchez R., Cabezón I., Boix-Palop L., Díaz-Pollán B., Goikoetxea A.J., García-País M.J., Pérez-Rodríguez M.T. (2025). Ceftaroline for bloodstream infections caused by methicillin-resistant *Staphylococcus aureus*: A multicentre retrospective cohort study. Clin. Microbiol. Infect..

[26] Hammond J., Benigno M., Bleibdrey N., Ansari W., Nguyen J.L. (2024). Ceftaroline fosamil for the treatment of methicillin-resistant *Staphylococcus aureus* bacteremia: A real-world comparative clinical outcomes study. Drugs Real. World Outcomes..

[27] Liu C., Bayer A., Cosgrove S.E., Daum R.S., Fridkin S.K., Gorwitz R.J., Kaplan S.L., Karchmer A.W., Levine D.P., Murray B.E. (2011). Clinical practice guidelines by the Infectious Diseases Society of America for the treatment of methicillin-resistant *Staphylococcus aureus* infections in adults and children. Clin. Infect. Dis..

[28] Haynes A.S., Maples H., Parker S. (2023). Time for a change: Considering vancomycin alternatives for pediatric methicillin-resistant *Staphylococcus aureus* bacteremia. J. Pediatric Infect. Dis. Soc..

[29] Rodvold K.A., McConeghy K.W. (2014). Methicillin-resistant *Staphylococcus aureus* therapy: Past, present, and future. Clin. Infect. Dis..

[30] Riccobene T.A., Khariton T., Knebel W., Das S., Li J., Jandourek A., Carrothers T.J., Bradley J.S. (2017). Population PK modeling and target attainment simulations to support dosing of ceftaroline fosamil in pediatric patients with acute bacterial skin and skin structure infections and community-acquired pneumonia. J. Clin. Pharmacol..

[31] Chan P.L.S., McFadyen L., Quaye A., Leister-Tebbe H., Hendrick V.M., Hammond J., Raber S. (2021). The use of extrapolation based on modeling and simulation to support high-dose regimens of ceftaroline fosamil in pediatric patients with complicated skin and soft-tissue infections. CPT Pharmacometrics Syst. Pharmacol..

[32] Korczowski B., Antadze T., Giorgobiani M., Stryjewski M.E., Jandourek A., Smith A., O’nEal T., Bradley J.S. (2016). A multicenter, randomized, observer-blinded, active-controlled study to evaluate the safety and efficacy of ceftaroline versus comparator in pediatric patients with acute bacterial skin and skin structure infection. Pediatr. Infect. Dis. J..

[33] Blumer J.L., Ghonghadze T., Cannavino C., O’nEal T., Jandourek A., Friedland H.D., Bradley J.S. (2016). A multicenter, randomized, observer-blinded, active-controlled study evaluating the safety and effectiveness of ceftaroline compared with ceftriaxone plus vancomycin in pediatric patients with complicated community-acquired bacterial pneumonia. Pediatr. Infect. Dis. J..

[34] Cannavino C.R., Nemeth A., Korczowski B., Bradley J.S., O’nEal T., Jandourek A., Friedland H.D., Kaplan S.L. (2016). A randomized, prospective study of pediatric patients with community-acquired pneumonia treated with ceftaroline versus ceftriaxone. Pediatr. Infect. Dis. J..

[35] Coella R., Glynn J.R., Gaspar C., Picazo J.J., Fereres J. (1997). Risk factors for developing clinical infection with methicillin-resistant *Staphylococcus aureus* (MRSA) amongst hospital patients initially only colonized with MRSA. J. Hosp. Infect..

[36] Sadoyama G., Gontijo Filho P.P. (2000). Risk factors for methicillin-resistant and sensitive *Staphylococcus aureus* infection in a Brazilian university hospital. Braz. J. Infect. Dis..

[37] Epstein L., Me Y., Belflower R., Scott J., Ray S., Dumyati G., Felsen C., Petit S., Yousey-Hindes K., Nadle J. (2015). Risk factors for invasive methicillin-resistant *Staphylococcus aureus* infection after recent discharge from an acute care hospitalization, 2011–2013. Clin. Infect. Dis..

[38] Tsai C.-E., Yang C.-J., Chuang Y.-C., Wang J.-T., Sheng W.-H., Chen Y.-C., Chang S.-C. (2022). Evaluation of the synergistic effect of ceftaroline against methicillin-resistant *Staphylococcus aureus*. Int. J. Infect. Dis..

[39] Barber K.E., Werth B.J., Rybak M.J. (2015). The combination of ceftaroline plus daptomycin allows for therapeutic de-escalation and daptomycin sparing against MRSA. J. Antimicrob. Chemother..

